# Supplementary material for: Empowered mothers and co-resident grandmothers: Two fundamental roles of women impacting child health outcomes in Punjab, Pakistan
Source: PLoS One. 2023 Nov 3;18(11):e0285995. doi: 10.1371/journal.pone.0285995 (PMC10624287; doi:10.1371/journal.pone.0285995)
Supplement: S1 Table — (PDF) [file pone.0285995.s001.pdf]

S1 Table A1: Measuring the Impact of Mother's empowerment on the Child Nutritional Outcomes using PDHS

	Additive Index		MCA Index	
VARIABLES	Weight For Age Z-Scores	Height for Age Z-Scores	Weight For Age Z-Scores	Height for Age Z-Scores
Mother's Empowerment	0.298**	0.181	1.168*	0.711
	(0.145)	(0.133)	(0.611)	(0.549)
Child's age	-0.0526***	-0.00731	-0.053***	-0.007
	(0.0108)	(0.0113)	(0.011)	(0.011)
Child's age Squared	0.347	0.627*	0.884	0.979*
	(0.612)	(0.368)	(0.804)	(0.523)
gender=1 if the child is a boy	-0.0103	-0.0667**	-0.010	-0.067**
	(0.0346)	(0.0291)	(0.035)	(0.030)
Dummy=1 if urban	-0.0805	-0.131	-0.081	-0.131
	(0.143)	(0.134)	(0.145)	(0.136)
Number of Household Members	0.00577	0.00374	0.006	0.004
	(0.0101)	(0.00840)	(0.010)	(0.009)
Dummy=1 if mother is currently working	-0.196**	-0.0795	-0.196*	-0.080
	(0.0994)	(0.0861)	(0.101)	(0.087)
Mother's age	-0.0769*	-0.00519	-0.0812*	-0.00519
	(0.04717)	(0.03217)	(0.0562)	(0.03217)
Mother's age squared	0.00128**	0.000228**	0.00128**	0.000228**
	(0.000634	(0.0000534	(0.000634	(0.0000534
Mother's Education	0.0159***	0.0140***	0.016***	0.014***
	(0.00753)	(0.00577)	(0.0077)	(0.0059)
age of Mother at 1st birth	-0.00960	-0.00542	-0.010	-0.005
	(0.00967)	(0.00797)	(0.010)	(0.008)
Dummy=1 if Mother is Currently breastfeeding	-0.123**	-0.0622	-0.123**	-0.062
	(0.0513)	(0.0456)	(0.052)	(0.046)
Dummy=1 if HH is in the first wealth Quintile	-0.148	-0.127	0.000	-0.127
	(0.206)	(0.192)	(0.000)	(0.195)
Dummy=1 if the HH is in second wealth Quintile	-0.222*	-0.193*	-0.075	-0.193*
	(0.122)	(0.115)	(0.143)	(0.117)
Dummy=1 if the HH is in third wealth Quintile	-0.0764	-0.111	0.071	-0.111
	(0.120)	(0.103)	(0.146)	(0.105)
Dummy=1 if HH is in the fourth wealth Quantile	-0.198**	-0.165**	-0.050	-0.165**
	(0.0962)	(0.0808)	(0.210)	(0.082)
Constant	0.307	0.691**	1.704*	1.346*
	(0.467)	(0.321)	(0.944)	(0.719)
Observations	4,604	5,156	4,604	5,156

Note: The fifth Wealth Quintile is omitted category
Standard errors are in parentheses *** p<0.01, ** p<0.05, * p<0.1
