# Supplementary material for: Empowered mothers and co-resident grandmothers: Two fundamental roles of women impacting child health outcomes in Punjab, Pakistan
Source: PLoS One. 2023 Nov 3;18(11):e0285995. doi: 10.1371/journal.pone.0285995 (PMC10624287; doi:10.1371/journal.pone.0285995)
Supplement: S2 Table — (PDF) [file pone.0285995.s002.pdf]

S2 Table A2: Measuring the Impact of the presence of grandmother on the nutritional outcomes of the child in a household using MICS

Dependent variables	Weight For Age Z-Scores	Height For Age Z-Scores
Dummy=1 if only Grandmother is present	0.0984**	0.0823
	(0.0451)	(0.0548)
Dummy=1 if the child is a girl	0.0428***	0.0320*
	(0.0484)	(0.0180)
	(0.0148)	(0.0160)
Age of the Child	-0.0767***	-0.117***
	(0.0132)	(0.00299)
Child's Age Squared	0.0196***	0.133***
	(0.00246)	(0.0116)
Dummy=1 if HH lives in urban	0.167***	0.00578
	(0.00958)	(0.0210)
Dummy=1 if the household head is a female	0.0201	0.00256*
	(0.0174)	(0.00133)
Total Number of Households	0.00344***	0.278***
	(0.00110)	(0.00740)
Wealth Score	0.254***	0.0555***
	(0.00610)	(0.00381)
Wealth Score Squared	0.0226***	0.112***
	(0.00338)	(0.00448)
Household head Education Level	0.0314***	0.00840**
	(0.00314)	(0.00410)
Mother's education level	0.0920***	-0.145***
	(0.00370)	(0.0194)
Mother's Age	-0.0428***	0.00110***
	(0.0160)	(0.000269)
Mother's Age Square	0.000876***	0.00371
	(0.000222)	(0.00263)
Age of the first born child	0.00271	0.00346
	(0.00216)	(0.00237)
Number of years Mother is married	0.00259	0.0129
	(0.00195)	(0.0145)
Dummy=1 if the mother currently breast feed	0.111***	-0.0571***
	(0.0120)	(0.0200)
Dummy=1 if Mother received Post-natal Care	-0.0289*	0.00192
	(0.0166)	(0.0214)
Dummy=1 if mother used contraceptive ever	-0.00903	-0.0478***
	(0.0176)	(0.0145)
Dummy=1 if the mother is currently pregnant	-0.0325***	0.0172
	(0.0120)	(0.0122)
Dummy=1 if the child got vaccination	-0.00548	-0.00519
	(0.0101)	(0.0101)
Mothers age at the first birth	0.0877***	-0.00203***
	(0.0158)	(0.000286)
Mothers age at the first birth squared	-0.00156***	-0.171***
	(0.000235)	(0.0186)
Families with 2 or more children	-0.138***	-0.319***
	(0.0153)	(0.0193)
Families with 3 or more children	-0.229***	-0.0123
	(0.0159)	(0.0259)
Constant	-119.7***	-59.44***
	(4.304)	(5.227)
Observations	99,218	98,229
R-squared	0.114	0.125

Note: Standard errors are in parentheses
*** p<0.01, ** p<0.05, * p<0.1
