# Supplementary material for: Empowered mothers and co-resident grandmothers: Two fundamental roles of women impacting child health outcomes in Punjab, Pakistan
Source: PLoS One. 2023 Nov 3;18(11):e0285995. doi: 10.1371/journal.pone.0285995 (PMC10624287; doi:10.1371/journal.pone.0285995)
Supplement: S3 Table — (PDF) [file pone.0285995.s003.pdf]

S3 Table B: Robustness checks Second-stage results with different cutoffs

Dependent Variable: WFA	Age cut off at 50 years	Age cutoff at 55 years	Age cut off at 60 years	Age cut off at 65 years	Age cut off at 70 years
Presence of Grandmother in a HH	0.0909**	0.0984**	0.121*	2.519	0.0288
	(0.0394)	(0.0451)	(0.0726)	(4.277)	(0.0972)
Observations	99,218	99,218	99,218	99,218	99,218
R-squared	0.114	0.114	0.114	0.001	0.114

Standard errors in parentheses *** p<0.01, ** p<0.05, * p<0.1
