# Supplementary material for: Empowered mothers and co-resident grandmothers: Two fundamental roles of women impacting child health outcomes in Punjab, Pakistan
Source: PLoS One. 2023 Nov 3;18(11):e0285995. doi: 10.1371/journal.pone.0285995 (PMC10624287; doi:10.1371/journal.pone.0285995)
Supplement: S4 Table — (PDF) [file pone.0285995.s004.pdf]

S4 Table C. The results for the Hausman test are shown below:

Dependent Variable: Weight for Age Z-Scores	
Fitted values (Mother's empowerment)	1.872***
	(0.674)
v	0.00648
	(0.00517)
Constant	-6.392***
	(2.211)
Observations	4,604
R-squared	0.265
F-test	10.489

Author's Calculations.

Note: The coefficient associated with the mother's empowerment under this setting can be different from the results reported in the final results reported in the paper, since the entire procedure is done manually, after running first and second stages separately and specification controls for and extra term v (predicted error from the first stage). The error v from the first-stage is insignificant implying that the error from the first stage and dependent variable of the second stage are uncorrelated.
