# Supplementary figures and images for: Empowered mothers and co-resident grandmothers: Two fundamental roles of women impacting child health outcomes in Punjab, Pakistan
Source: PLoS One. 2023 Nov 3;18(11):e0285995. doi: 10.1371/journal.pone.0285995 (PMC10624287; doi:10.1371/journal.pone.0285995)

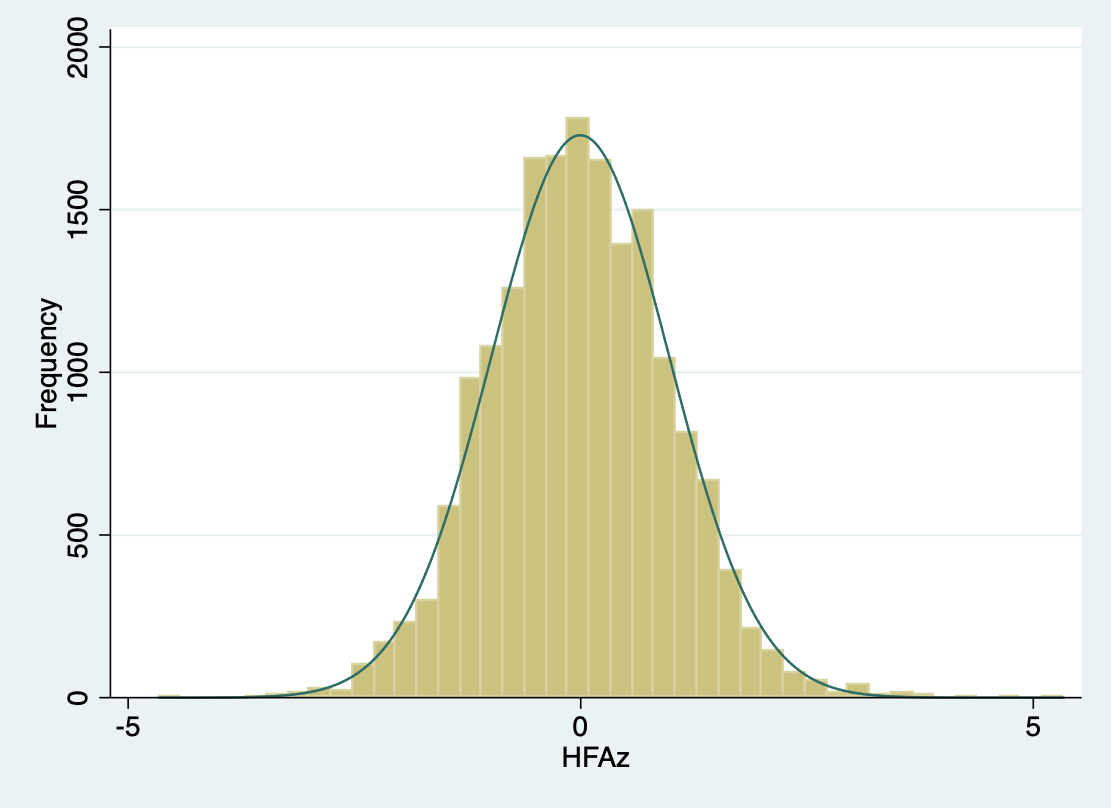

Supplement: S1 Fig — (TIF) [file pone.0285995.s005.tif]

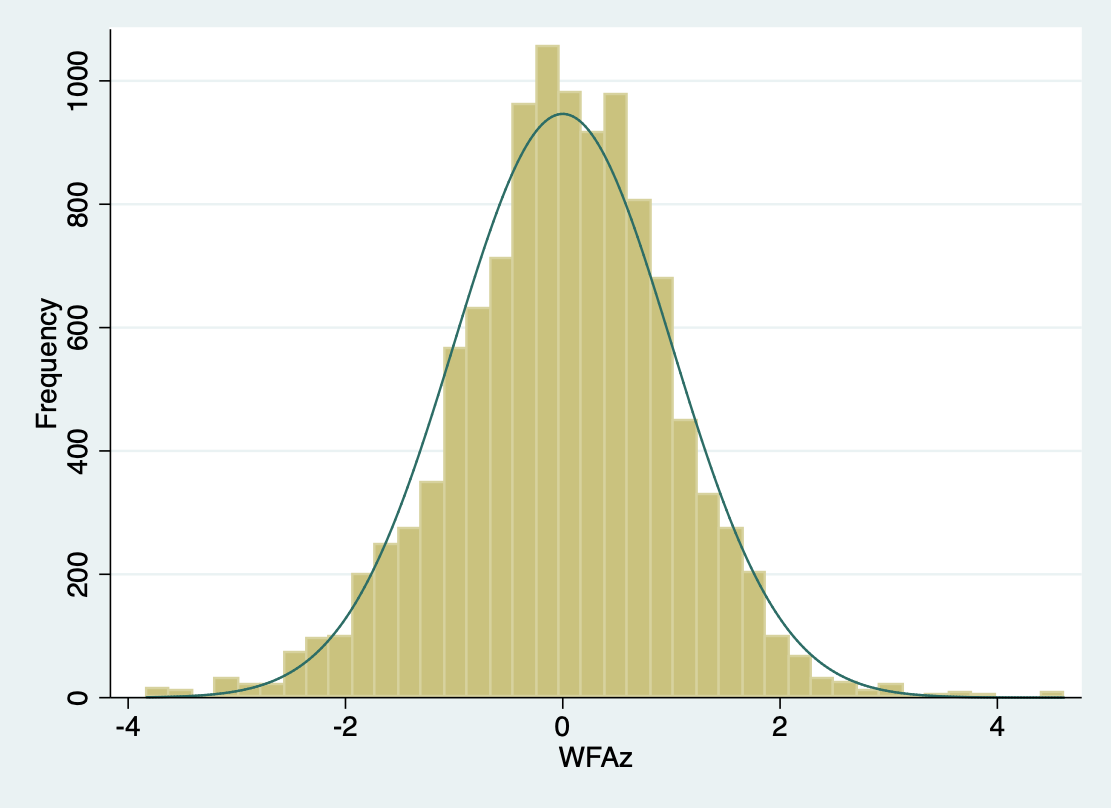

Supplement: S2 Fig — (TIF) [file pone.0285995.s006.tif]

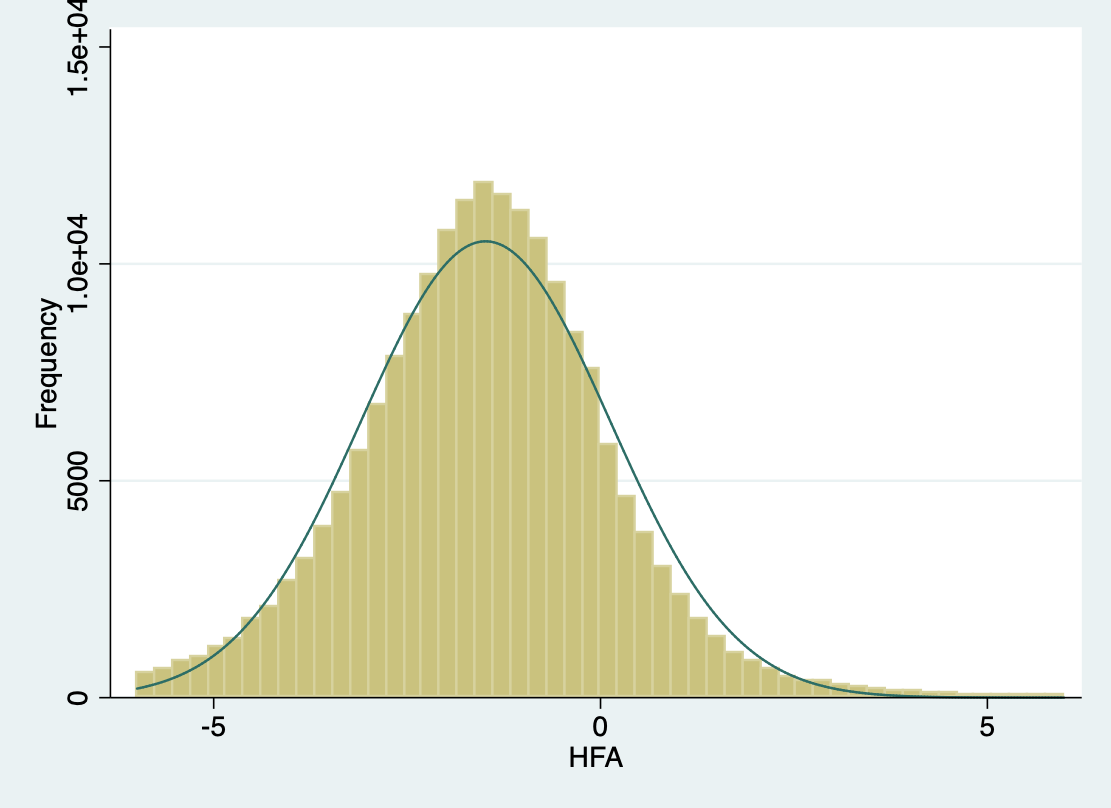

Supplement: S3 Fig — (TIF) [file pone.0285995.s007.tif]

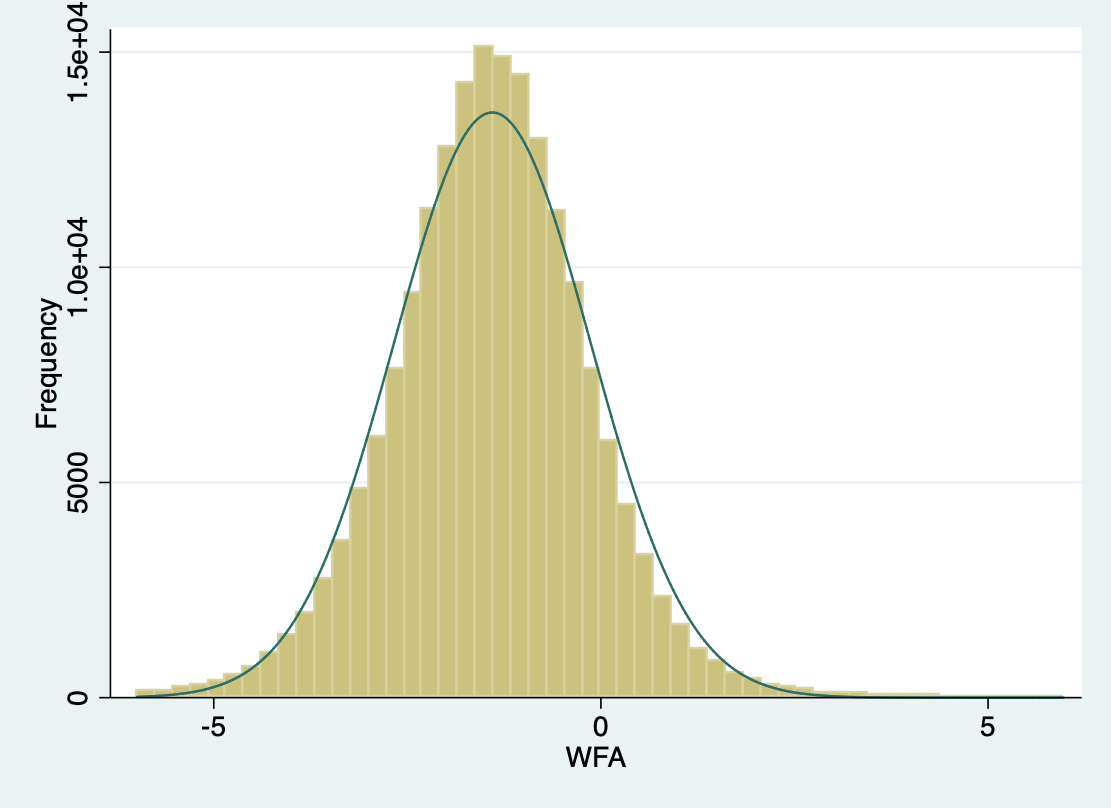

Supplement: S4 Fig — (TIF) [file pone.0285995.s008.tif]
